# Using Medical Student Quality Improvement Projects to Promote Evidence-Based Care in the Emergency Department

**DOI:** 10.5811/westjem.2017.9.35163

**Published:** 2017-12-05

**Authors:** Michael W. Manning, Eric W. Bean, Andrew C. Miller, Suzanne J. Templer, Richard S. Mackenzie, David M. Richardson, Kristin A. Bresnan, Marna R. Greenberg

**Affiliations:** * University of South Florida College of Medicine, Department of Emergency Medicine, Tampa, Florida; † University of South Florida College of Medicine, Department of Internal Medicine, Tampa, Florida; ‡ University of South Florida College of Medicine, Department of Family Medicine, Tampa, Florida; § Lehigh Valley Hospital, Department of Emergency Medicine, Allentown, Pennsylvania; ¶ Lehigh Valley Hospital, Department of Internal Medicine, Allentown, Pennsylvania; || Lehigh Valley Hospital, Department of Family Medicine, Allentown, Pennsylvania

## Abstract

**Introduction:**

The Association of American Medical Colleges’ (AAMC) initiative for Core Entrustable Professional Activities for Entering Residency includes as an element of Entrustable Professional Activity 13 to “identify system failures and contribute to a culture of safety and improvement.” We set out to determine the feasibility of using medical students’ action learning projects (ALPs) to expedite implementation of evidence-based pathways for three common patient diagnoses in the emergency department (ED) setting (Atrial fibrillation, congestive heart failure, and pulmonary embolism).

**Methods:**

These prospective quality improvement (QI) initiatives were performed over six months in three Northeastern PA hospitals. Emergency physician mentors were recruited to facilitate a QI experience for third-year medical students for each project. Six students were assigned to each mentor and given class time and network infrastructure support (information technology, consultant experts in lean management) to work on their projects. Students had access to background network data that revealed potential for improvement in disposition (home) for patients.

**Results:**

Under the leadership of their mentors, students accomplished standard QI processes such as performing the background literature search and assessing key stakeholders’ positions that were involved in the respective patient’s care. Students effectively developed flow diagrams, computer aids for clinicians and educational programs, and participated in recruiting champions for the new practice standard. They met with other departmental clinicians to determine barriers to implementation and used this feedback to help set specific parameters to make clinicians more comfortable with the changes in practice that were recommended. All three clinical practice guidelines were initiated at consummation of the students’ projects. After implementation, 86% (38/44) of queried ED providers felt comfortable with medical students being a part of future ED QI initiatives, and 84% (26/31) of the providers who recalled communicating with students on these projects felt they were effective.

**Conclusion:**

Using this novel technique of aligning small groups of medical students with seasoned mentors, it is feasible for medical students to learn important aspects of QI implementation and allows for their engagement to more efficiently move evidence-based medicine from the literature to the bedside.

## BACKGROUND

Quality improvement (QI) initiatives to advance patient care have become widespread in healthcare.^1^ The healthcare industry has adapted many “change” implementation tools from other industries, such as lean management, six sigma, and more recently bidirectional alignment.^1^ Bidirectional alignment is the idea that an institutional problem should be evaluated and addressed from the bottom-up as well as the top-down.^2^ This means giving the people on the frontline of care a voice in the ivory tower of organizational priority setting.^3^ The Association of American Medical Colleges’ (AAMC) initiative for Core Entrustable Professional Activities for Entering Residency includes as an element of Entrustable Professional Activity 13 to “identify system failures and contribute to a culture of safety and improvement.”^4^ The AAMC Aligning and Educating Quality Initiative has the aspiration of “aligning and educating for quality to assist medical schools in development of curriculum, faculty, and programs in systematic incorporation of these skills starting in the earliest stages of medical careers.^5^

We designed three educational innovations that stressed bidirectional alignment by pairing senior practitioners in the emergency department (ED) with teams of six third-year medical students on three separate QI initiatives designed to develop clinical pathways for congestive heart failure, pulmonary embolism, and atrial fibrillation. The rationale for such an approach is to pair the experience and knowledge of veteran practitioners with the learning mindset and fresh perspective of medical students to create novel solutions, thus improving quality of care.^6^ Involving future-oriented learners in the processes of QI better enables organizations to proactively adapt to continually evolving regulations vs. reacting from the top-down with entrenched approaches to meeting standards of care.^7^

## OBJECTIVES

We set out to determine the feasibility of using third-year medical students’ action learning projects (QI projects) to expedite implementation of evidence-based pathways for three common patient diagnoses in the ED setting as well as develop a model for promoting bidirectional alignment at an institutional level. We further evaluated clinician perspectives on using medical students at the forefront of QI pathway development.

## CURRICULAR DESIGN

These prospective QI initiatives were performed over six months in three Northeastern PA hospitals. One was a Level I trauma center with an annual census of 90,000, one was a suburban hospital with an annual census of 60,000, and the third was an inner city hospital with an annual census of over 32,000 visits per year. Emergency physician mentors were recruited by medical school faculty to facilitate a QI experience for third-year medical students for each project. These physician sponsor/mentors had no training to lead such QI teams, but all were established leaders in the ED who were familiar with teaching (core emergency medicine [EM] residency faculty) and had participated in QI initiatives previously (examples: the ED vice-chair of QI, a hospital site director, etc). Students were given class time and network infrastructure support (including information technology and consultant experts in lean management) to develop, evaluate, and implement changes in clinical pathways. The timeline and detailed description of the program with faculty time estimates is provided in the table. The network institutional review board (IRB) reviewed the project and found it to be consistent with QI, and thus IRB oversight was not required.

**Population Health Research Capsule**

What do we already know about this issue?

The AAMC Aligning and Educating Quality Initiative has the aspiration of encouraging the development of curriculum for quality improvement skills in the early stages of medical careers.

What was the research question?

Can third year medical students help expedite implementation of evidence-based pathways for common patient diagnoses in the emergency department (ED).

What was the major finding of the study?

Using bidirectional alignment of medical students with mentors, it is feasible for students to learn important aspects of QI.

How does this improve population health?

Using medical students to help promote evidence-based care in the ED indirectly improves population health.

Students had access to background network data that revealed potential for improvement in disposition (to home) for patients with all three diagnoses. Each group was expected to use lean management tools, such as plan-do-study-act, to develop a root-cause fishbone diagram to determine the current state, use A3 problem solving, determine and engage the key stakeholders, develop a clinical pathway, recruit champions for change management, and establish a plan for measuring the outcomes of their respective QI initiatives. Following conclusion of the initiatives the students were required to present their projects to peers and the key stakeholders.

The educational methods described were chosen to facilitate bidirectional engagement of senior providers and medical students in order to have a meaningful, team-based impact on QI initiatives in the ED.^8^ This provided students with the opportunity to learn how clinical practices are evaluated and improved at an institutional level, while enabling the many strata of providers at the clinical frontline to coordinate their efforts to improve quality of care. The students and providers noted challenges when it came to engaging in change management in a hectic and distracting work environment, reconciling multiple pathways from interdisciplinary feedback, and obtaining sufficient data from the electronic medical records. At the conclusion of the projects clinical providers were surveyed regarding their level of comfort with medical students being involved in future QI initiatives in the ED, as well as whether medical student involvement affected their likelihood of using the proposed clinical pathways.

## IMPACT/EFFECTIVENESS

**Figure 1:**
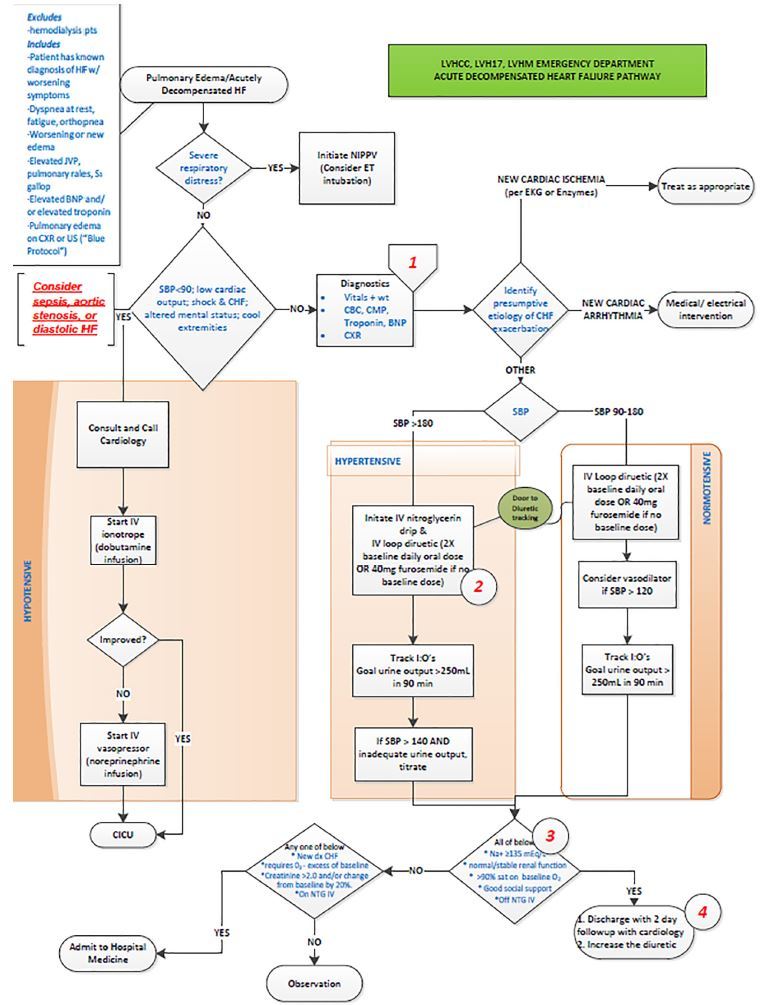
Congestive heart failure pathway.

**Figure 2:**
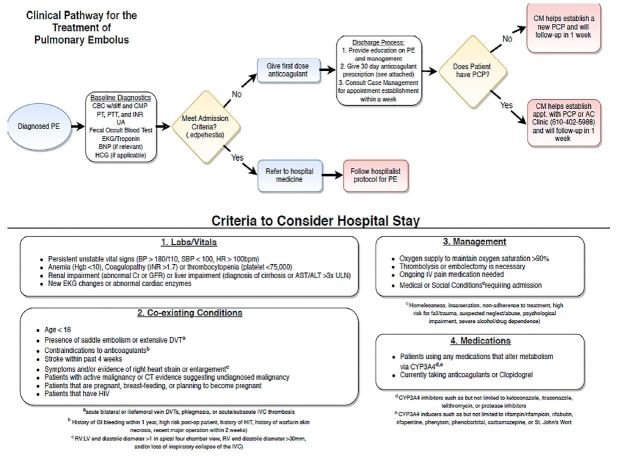
Pulmonary embolism pathway.

**Figure 3:**
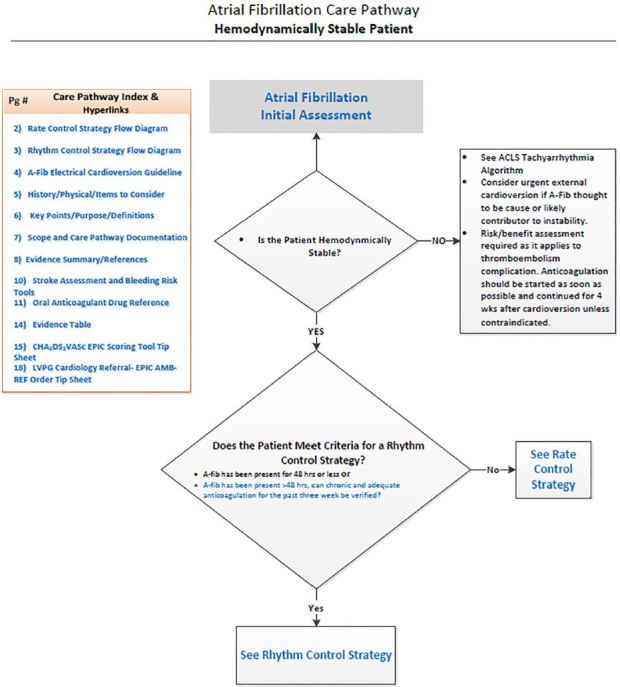

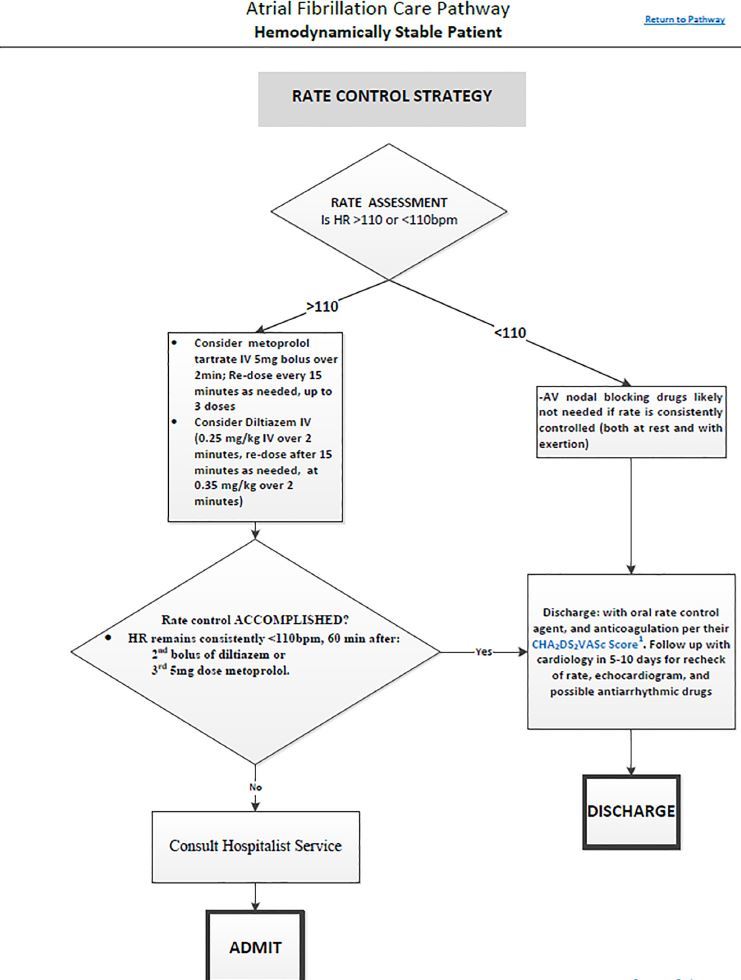

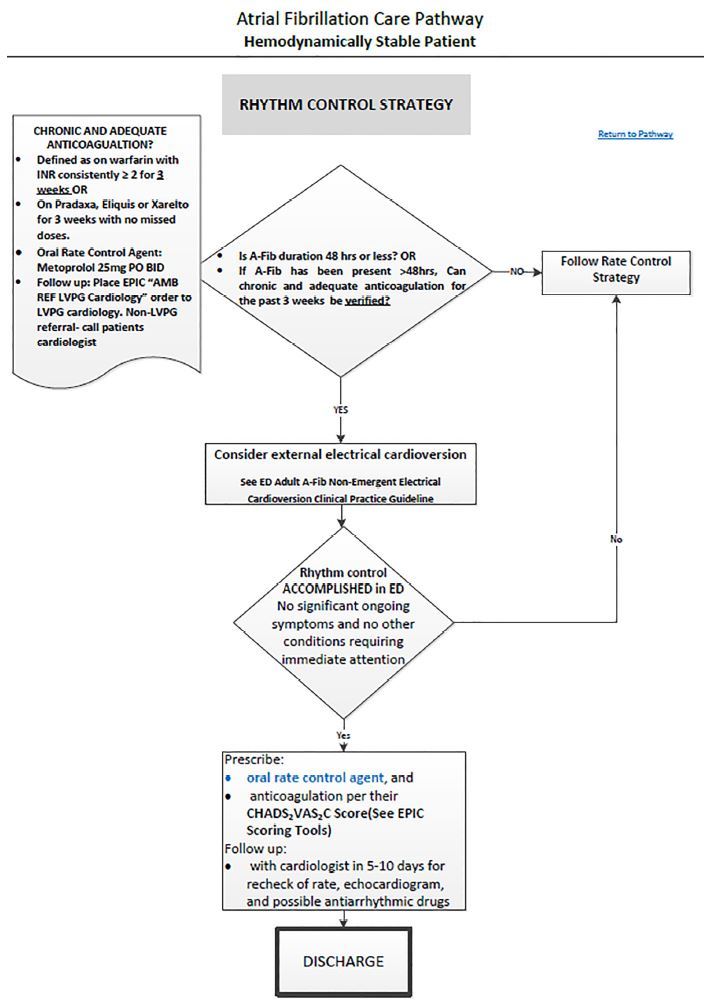

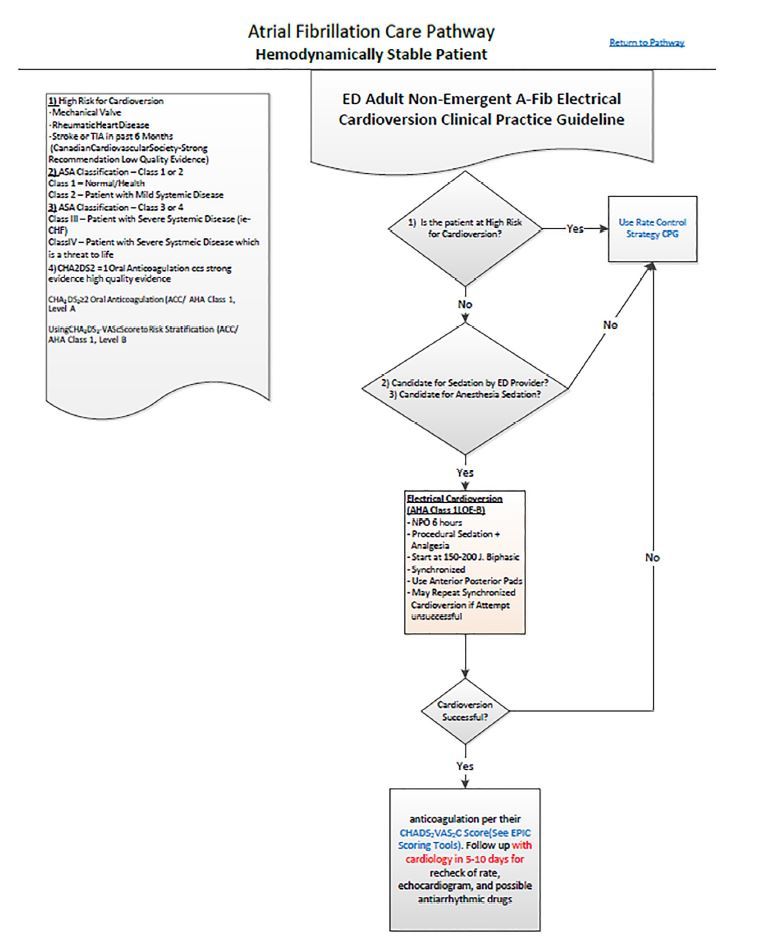
Atrial fibrillation pathway.

Under the leadership of their mentors, students accomplished standard QI processes such as performing the background literature search, assessing key stakeholders’ positions with respect to patient care, and metrics for measuring success. Students effectively developed flow diagrams, computer aids for clinicians, educational programs and participated in recruiting champions for the new practice standard. They met with other departmental clinicians to determine barriers to implementation and used this feedback to help set specific parameters to make clinicians more comfortable with the changes in practice that were recommended. All three clinical practice guidelines (Figures 1–3) were initiated in an orchestrated manner at the consummation of the students’ projects. These guidelines have been implemented for approximately six months.

After implementation, 86% (38/44) of the departmental providers felt comfortable with medical students being a part of future ED QI initiatives. Eighty-four percent (26/31) of the providers who recalled communicating with students on these projects (for example as a champion, as a clinician using the pathway, or having received education from the students) felt they were effective. The majority (66%) of providers surveyed felt that using medical students for developing these pathways did not affect their attitude on whether they would use the pathway in clinical practice. Only three providers surveyed felt that using medical students for developing these pathways would make it less likely they would use the pathway.

To date, this curriculum is limited by the fact that we do not have statistical outcome measures to report. However, since initiation, the network and ED QI committees following the implementation of these pathways have had no patient adverse or serious events to report. Additionally, the action learning project described is a curriculum portion of a program for medical students (USF SELECT). The SELECT program has faculty trained in lean methodology and leadership. These resources were available to our network without additional expense or training, although their faculty time is included in the model (Table 1). Whether this training is generalizable to other networks that may not have this robust availability of both medical student faculty or QI infrastructure is unclear.

Using this novel technique of bidirectional alignment of small groups of medical students with seasoned mentors, it is feasible for medical students to learn important aspects of QI implementation and allows for their engagement to more efficiently move evidence-based medicine from the literature to the bedside. Further study with data outcomes to illustrate consistency of algorithm use is needed.

## Figures and Tables

**Table 1:** Timeline and training for action learning projects.

Task	Total hours	Faculty	Mentor/sponsor	Student	Start date	End date
Identify project sponsors/mentors	4	4	1	0	9/1/2016	10/5/2016
Team development (students receive class training), projects are assigned and ALP starts	3	3	0	3	10/06/2016	10/06/2016
Project management (students receive class training, develop guiding principles, appoint a project leader and manager)	4	4	0	4	11/3 and 1/12/2017	1/12/2017
ALP Group class and/or workgroup time (students have class time to work on projects together) Meet with project sponsor, complete project structure, scope project, draft responsibility chart, develop A3	20	0	20	20	10/6/2016	4/27/2016
Prepare Project Report Out	2	0	0	2	4/27/2017	5/10/2017
Presentation skills (students receive class training)	1	1	0	1	4/27/2017	4/27/2017
ALP class presentations (students present to each other their projects)	4	4	2	4	5/11/2017	5/11/2017
Faculty evaluation of projects	3	3	0	0	5/11/2017	5/19/2017
Summary hours	41	19	23	34+	9/1/2016	5/19/2017

*ALP*, action learning project.

+ Exact estimate of time for students is not possible. Students had 34 hours of protected class time to complete this work, but the vast majority spent many more hours outside class researching their topics, connecting with champions, and doing departmental staff training.
